# Liver X receptors agonist promotes differentiation of rat bone marrow derived mesenchymal stem cells into dopaminergic neuron-like cells

**DOI:** 10.18632/oncotarget.23076

**Published:** 2017-12-09

**Authors:** Oumei Cheng, Xiaoyan Tian, Ying Luo, Shaoshan Mai, Yang Yang, Shengnan Kuang, Qi Chen, Jie Ma, Beibei Chen, Rong Li, Lu Yang, Huan Li, Congli Hu, Jiahua Zhang, Zhihao Chen, Yuke Li, Hui Xia, Ying Xu, Junqing Yang

**Affiliations:** ^1^ Department of Pharmacology, Chongqing Medical University, The Key Laboratory of Biochemistry and Molecular Pharmacology, Chongqing 400016, China; ^2^ Department of Neurology, The First Affiliated China Hospital, Chongqing Medical University, Chongqing, 400016, China; ^3^ Department of Pharmaceutical Sciences, School of Pharmacy and Pharmaceutical Sciences, State University of New York at Buffalo, Buffalo, NY 14214, USA

**Keywords:** Parkinson’s disease, dopaminergic neurons, liver X receptors, bone marrow derived mesenchymal stem cells and cell differentiation

## Abstract

Dopaminergic (DA) neurons derived from bone marrow derived mesenchymal stem cells (BMSCs) maybe a valuable source for cell replacement therapy in Parkinson disease. Recent studies showed that new functions of LXR and their ligands have been proposed to prevent PD in the adult nervous system. The present study was designed to observe the effect of liver X receptors (LXR) agonist on differentiation of rat BMSCs into DA neurons. Expressions of the neuronal markers (Tuj1 and Nestin), the specific marker of DA neurons (tyrosine hydroxylase, TH), LXR α and LXR β were measured by immunocytochemical assay and TH/Tuj1 positive cells were determined by quantitative cell count analyses. mRNA expressions of LXR α, LXR β, TH, DAT, Nurr1, Pitx3, En1 and Lmx1b were measured by qPCR. Compared with growth factors (GF) treated group, combined use of LXR and GF induced rat BMSCs to TH-expressing cells with 87.42% of efficiency in 6 days of period of induction. LXR agonist alone did not induce the differentiation. Compared with GF alone, combined use of LXR and GF increased expressions of LXR α and LXR β protein and mRNA and TH, DAT, Nurr1, and Pitx3 mRNA, decreased expressions of En1 and Lmx1b mRNA. Our experimental results indicated that LXR activation leads to improve induction efficiency and shorten induction period of rat BMSCs into DA neuron-like cells through regulating DA development-related genes expressions and that LXR can be considered as a candidate target for drug development to improve differentiation of BMSCs into DA neurons.

## INTRODUCTION

Parkinson’s disease (PD) is a chronic progressive neurodegenerative disease that affects over 1% of the population over 65 years of age and has been positioned as the second most common neurodegenerative disorder after Alzheimer’s disease [1]. It is characterized by a progressive and extensive loss of dopaminergic (DA) neurons in the substantia nigra pars compacta (SNpc) and their terminals in the striatum, which results in debilitating movement disorders. Currently available pharmacological and surgical therapies ameliorate clinical symptoms in the early stages of disease, but they cannot stop or reverse degeneration of DA neurons and have some serious side effects with long-term use [2]. Cell replacement therapy (CRT) offers great promise as the future of regenerative medicine in PD [3]. There are many reports about the derivation of DA neurons from embryonic stem cells (ESCs) [4, 5], neural stem cells (NSCs) [6] and mesenchymal stem cells (MSCs) [7]. In contrast to ESCs and NSCs, MSCs can be derived from the patient’s own bone marrow and easy to isolate. So, bone marrow derived MSCs (BMSCs) represent a potential source for autologous cell transplantation in avoiding or reducing immunological rejections. BMSCs usage for CRT circumvents the ethical problems concerning fetal tissue usage, thus makes itself attractive for regenerative medicine research [8]. Much effort has been laid to compare the effect of naive MSCs and MSCs differentiated into tyrosine hydroxylase (TH)-positive cells on transplanting in PD animal models. Although both naive and differentiated MSCs evoked behavioral recovery, the effect of the differentiated cells was more pronounced [2, 9–11].

Differentiation of BMSCs can be induced by exposure to growth factors (GF) [7], chemical inducers [12] and also by lentiviral transduction of DA transcription factors [13]. By means of *in vitro* manipulation with growth factors (sonic hedgehog and fibroblast growth factors, SHH and FGFs), Trzaska KA *et al.* succeeded in inducing adult human BMSCs to DA neurons with an 67% of efficiency in 12 days, which is the highest induction efficiency among the previous research reports [7]. However, the diverse differentiation methods, low production rate, and low survival rate after transplantation are still obstacles that need to be overcome before the application of BMSCs to treat PD [14] and the mechanisms involved in differentiation of BMSCs is not yet well understood.

The liver X receptors (LXR α and LXR β, encoded by *NR1H3* and *NR1H2*, respectively) are ligand-dependent transcription factors belonging to the nuclear hormone receptor superfamily [15, 16]. The most well-known functions of LXR are to regulate lipid metabolism and homeostasis [17, 18], increase cholesterol efflux from the cells and protect from cholesterol overload and toxicity [19]. LXR α/β double knockout mice showed a progressive accumulation of lipids in the brain and loss of adult spinal cord motor neurons and ventral midbrain (VM) DA neurons [20, 21]. These researches suggested that LXR is highly correlated to the development of DA neurons *in vivo*. However, it remains unknown whether LXR have effect on differentiation of adult rat BMSCs into DA neurons.

The present study was designed to investigate the effect of LXR agonist (TO901317) on the differentiation of rat BMSCs into DA neurons and its possible mechanism.

## RESULTS

### Characterization of rat BMSCs

**Figure 1:**
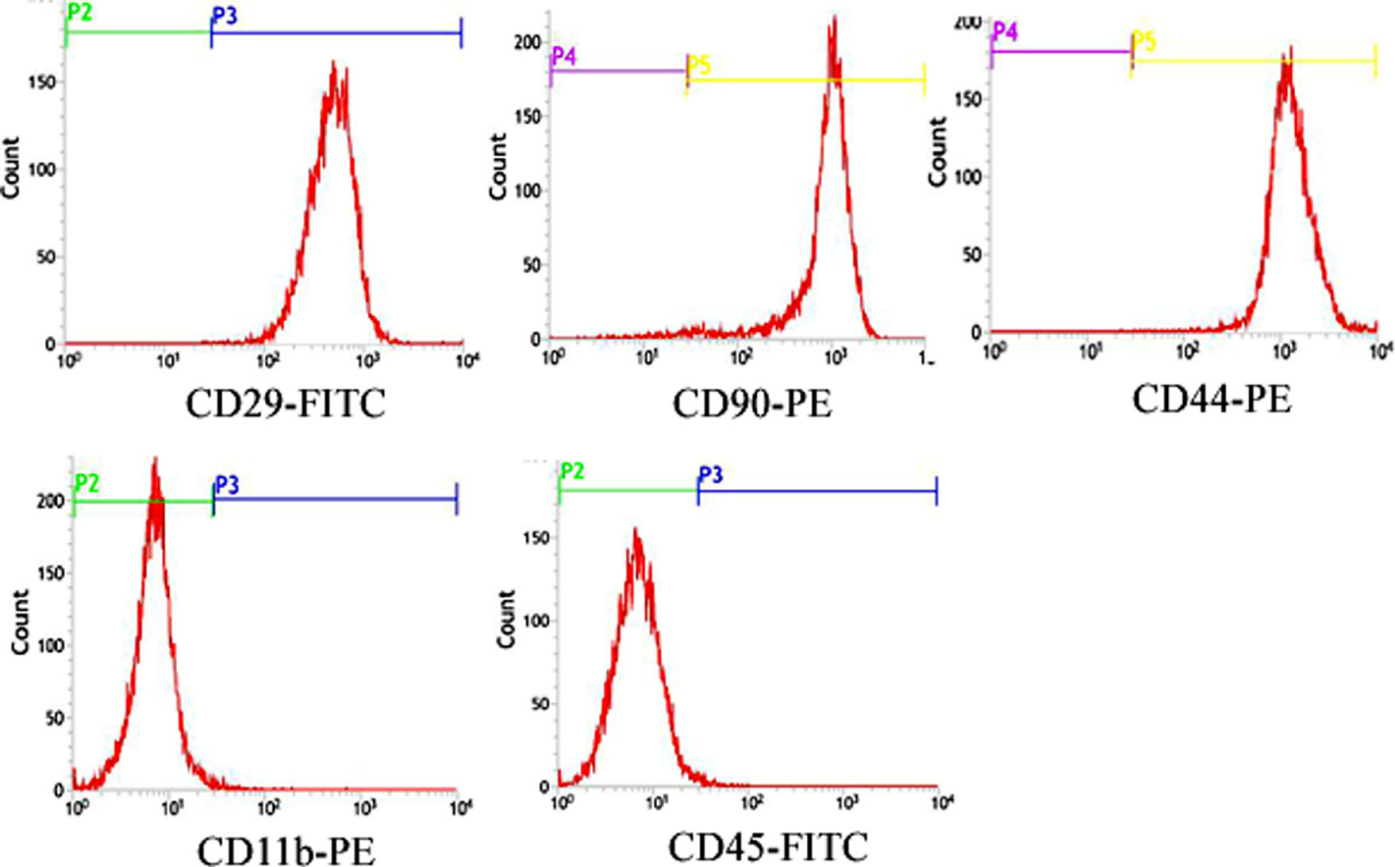
Characterization of rat BMSCs Flow Cytometric surface marker expression profile showed cells expressed CD29, CD44 and CD90 and were negative for CD45 and CD11b.

Undifferentiated rat BMSCs in expansion cultures were examined at 3rd passage for the expression of cell surface antigens by flow-cytometry, which commonly used in the characterization of rat BMSCs populations [22]. Rat BMSCs expressed CD29, CD44 and CD90 and were negative for CD45 and CD11b (Figure 1).

### Effects of combined use of LXR agonist with GF on differentiation of rat BMSCs into DA neurons

Immunocytochemistry was performed to evaluate the expressions of the neuronal markers (Tuj1 and Nestin) and the specific marker of DA neurons (TH). DA neurons are classically identified by expression of TH, the rate-limiting enzyme in the biosynthesis of DA [23].

### Determination for the adding time of LXR agonist

**Figure 2:**
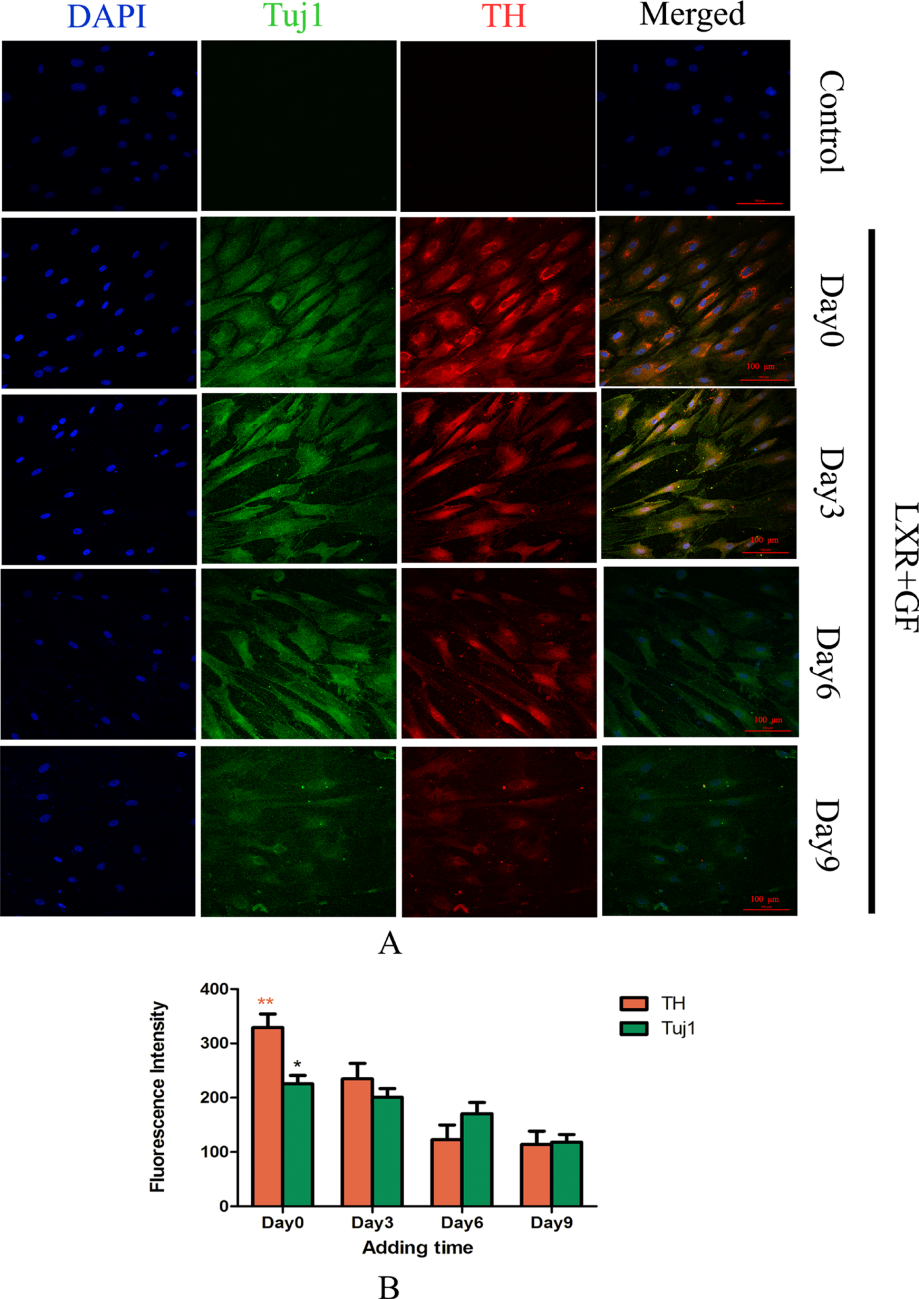
Determine the adding time of LXR agonist (× 200, Scale bars = 100 μm) (**A**) Change in expressions of Tuj1 and TH. No expression of Tuj1 and TH was detected in control group. Cells in LXR+GF group displayed merged images of double staining with Tuj1 (dylight 488, green) and TH (dylight 549, red). Colocalization was identified by the yellow fluorescence in the merged images. Expressions of Tuj1 and TH reached the peak at simultaneous addition of LXR agonist and GF (day 0). Accompanying with the adding time of LXR agonist delayed, expressions of Tuj1 and TH were decreased gradually. Thus, the optimal adding time of LXR agonist was at day 0. (**B**) Group data showing change in expressions of Tuj1 and TH. Data are expressed as mean ± SD (*n =* 5). ^*^P < 0.05 and ^**^P < 0.01, compared with day3, respectively.

Almost cells in control group probed with TH and Tuj1 antibodies were negative and only show the nuclear stain DAPI. Cells in LXR+GF group displayed merged images of double staining with Tuj1 (dylight 488, green) and TH (dylight 549, red). Colocalization was identified by the yellow fluorescence in the merged images. Expressions of Tuj1 and TH reached the peak at simultaneous addition of LXR agonist and GF (Day 0). Accompanying with the adding time of LXR agonist delayed, expressions of Tuj1 and TH were decreased gradually. Thus, the optimal adding time of LXR agonist was at Day 0 (Figure 2).

### Determination for induction period of LXR agonist

**Figure 3:**
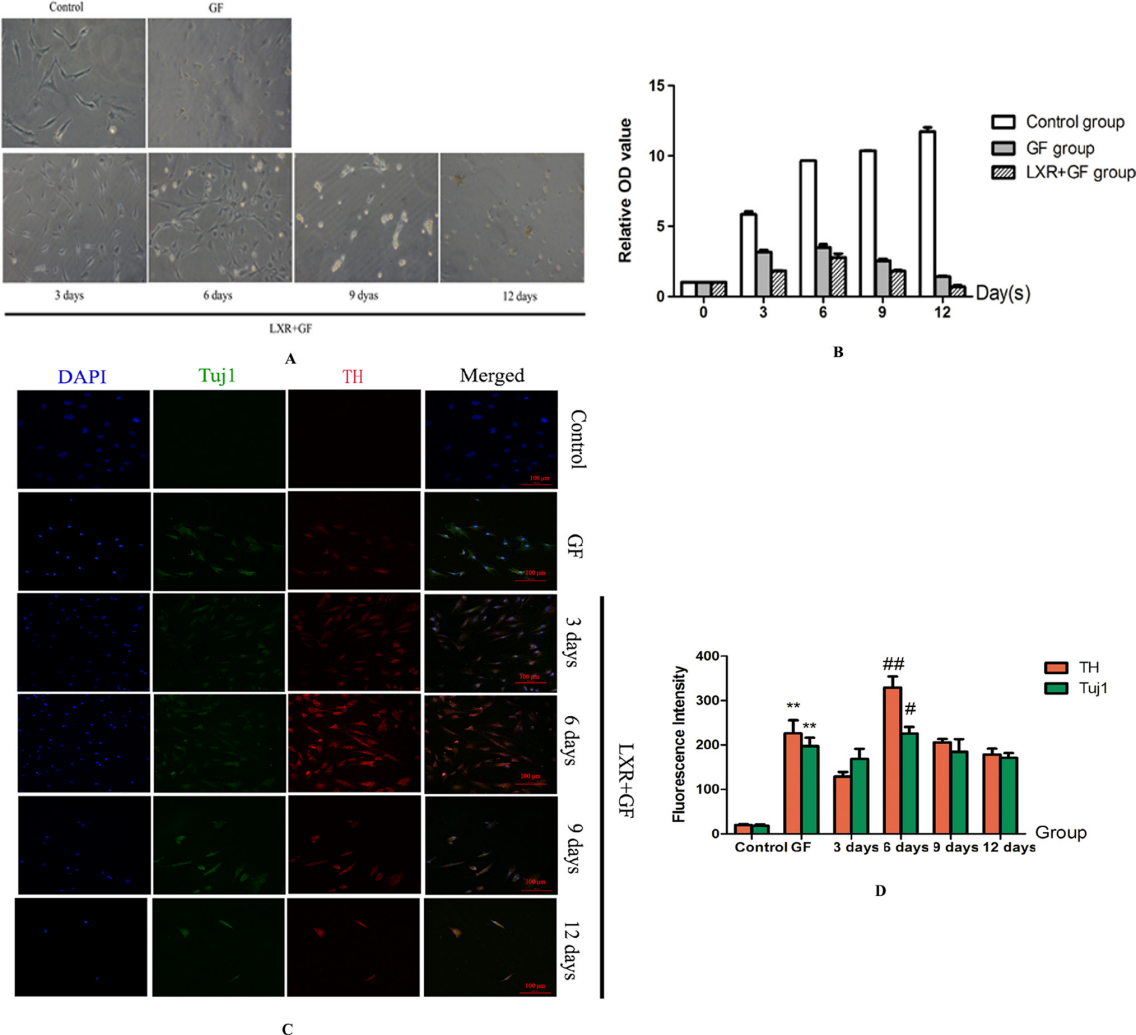
Determine the time for induction period of LXR agonist (× 200, Scale bars = 100 μm) (**A**) Bright-field images of rat BMSCs in different groups. Cells in GF group and LXR+GF (day 0) group revealed the appearance of extended long cellular processes and retracted cell bodies, which were the typical neuronal morphology, and the amount of cells in LXR+GF (day 0) group were increased and later decreased, and reached the peak at day 6 in the bright-field images. (**B**) The growth rate of cells in GF group and LXR+GF (day 0) group reached the peak at day 6 and the growth rate of cells in LXR+GF (day 0) group was consistent with images in the bright-field. (**C**) Change in expressions of Tuj1 and TH. No expression of Tuj1 and TH was detected in control group. Cells in GF group and LXR+GF group displayed merged images of double staining with Tuj1 (dylight 488, green) and TH (dylight 549, red). Colocalization was identified by the yellow fluorescence in the merged images. Expressions of Tuj1 and TH reached the peak at day 6 in LXR+GF group. Thus, the time for induction period of LXR agonist was 6 days. (**D**) Group data showing change in expressions of Tuj1 and TH. Data are expressed as mean ± SD (*n =* 5). ^**^P < 0.01, compared with control group. ^#^*P* < 0.05 and ^##^*P* < 0.01, compared with GF group, respectively.

Cells in GF group and LXR+GF (Day 0) group revealed the appearance of extended long cellular processes and retracted cell bodies, which were the typical neuronal morphology, and the amount of cells in LXR+GF (Day 0) group were increased and later decreased, and reached the peak at day 6 in the bright-field images (Figure 3A). The growth rate of cells in GF group and LXR+GF (Day 0) group reached the peak at day 6 and the growth rate of cells in LXR+GF (Day 0) group was consistent with images in the bright-field (Figure 3B).

**Figure 4:**
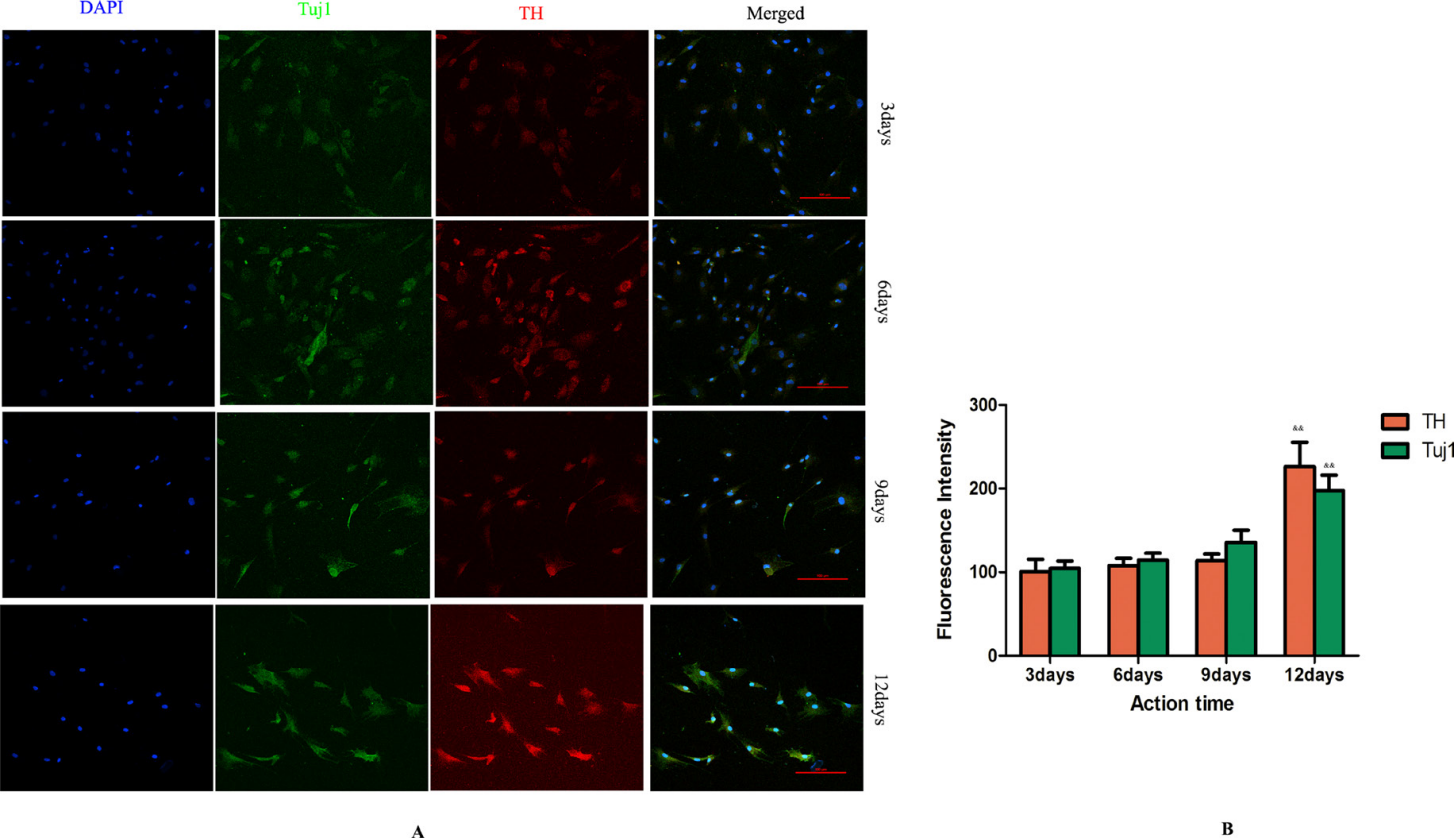
Determine the time for induction period of GF (× 200, Scale bars = 100 μm) (**A**) Change in expressions of Tuj1 and TH. Cells in GF group displayed merged images of double staining with Tuj1 (dylight 488, green) and TH (dylight 549, red). Colocalization was identified by the yellow fluorescence in the merged images. Expressions of Tuj1 and TH were gradually increased in GF group and maximal expressions were obtained at 12 days. (**B**) Group data showing change in expressions of Tuj1 and TH. Data are expressed as mean ± SD (*n =* 5). ^&&^*P* < 0.01, compared with 9 days for induction.

Cells in GF group and LXR+GF (Day 0) group displayed merged images of double staining with Tuj1 (dylight 488, green) and TH (dylight 549, red). Colocalization was identified by the yellow fluorescence in the merged images. Expressions of Tuj1 and TH were gradually increased in GF group and maximal expressions were obtained at 12 days (Figure 4). Thus, the time for induction period of GF was 12 days and GF group is GF-treated-12day group for short. Expressions of Tuj1 and TH reached the peak at day 6 in LXR+GF group. Thus, the time for induction period of LXR agonist was 6 days (Figure 3C&D) and it was increased significantly compared with those of GF group (^#^*P* < 0.05 and ^##^*P* < 0.01, respectively). Compared with control group, expressions of Tuj1 and TH were increased significantly in GF group (^**^P < 0.01).

### Determination for the concentration of LXR agonist

**Figure 5:**
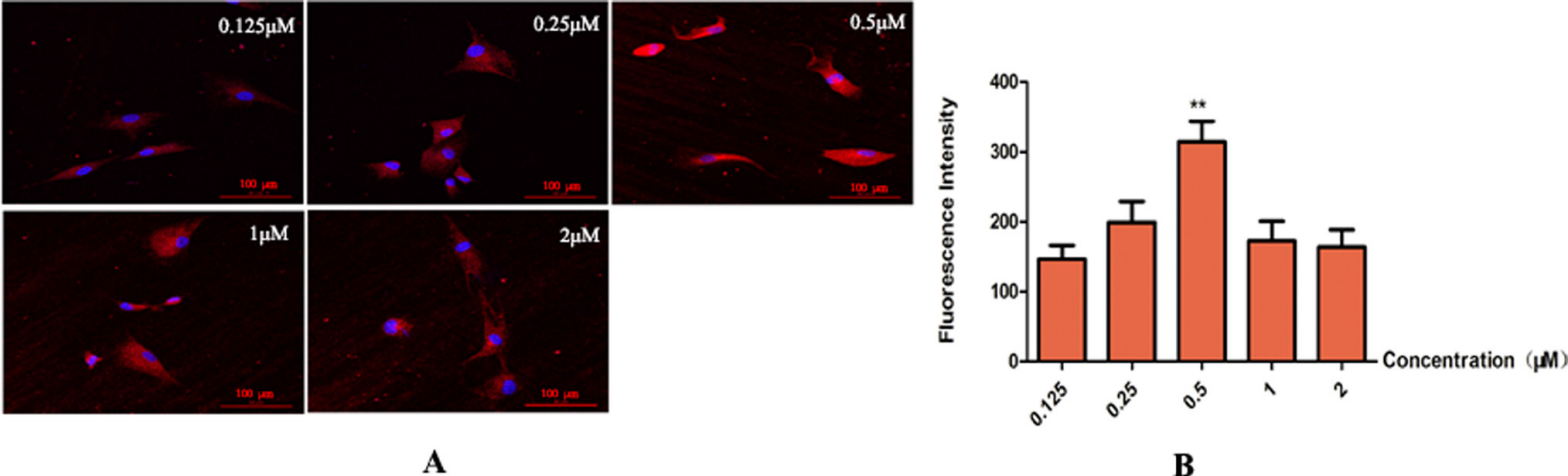
Determination for the concentration of LXR agonist (× 400, Scale bars = 50 μm) (**A**) Change in expression of TH. Maximal expression of TH was obtained at concentrations of 0.5 μM. Thus, 0.5 μM was determined as the concentration of LXR agonist. (**B**) Group data showing change in expression of TH. Data are expressed as mean ± SD (*n =* 5). ^**^P < 0.01, compared with concentration of 0.25 μM.

Five concentrations of LXR agonist (0.125, 0.25, 0.5, 1 and 2 μM) were used in the present study. Cells in different concentrations of LXR agonist showed the typical neuronal morphology. Maximal expression of TH was obtained at concentration of 0.5 μM (^**^P < 0.01). Thus, 0.5 μM was chosen as the concentration of LXR agonist in following experiments (Figure 5).

### Change in expression of Nestin

**Figure 6:**
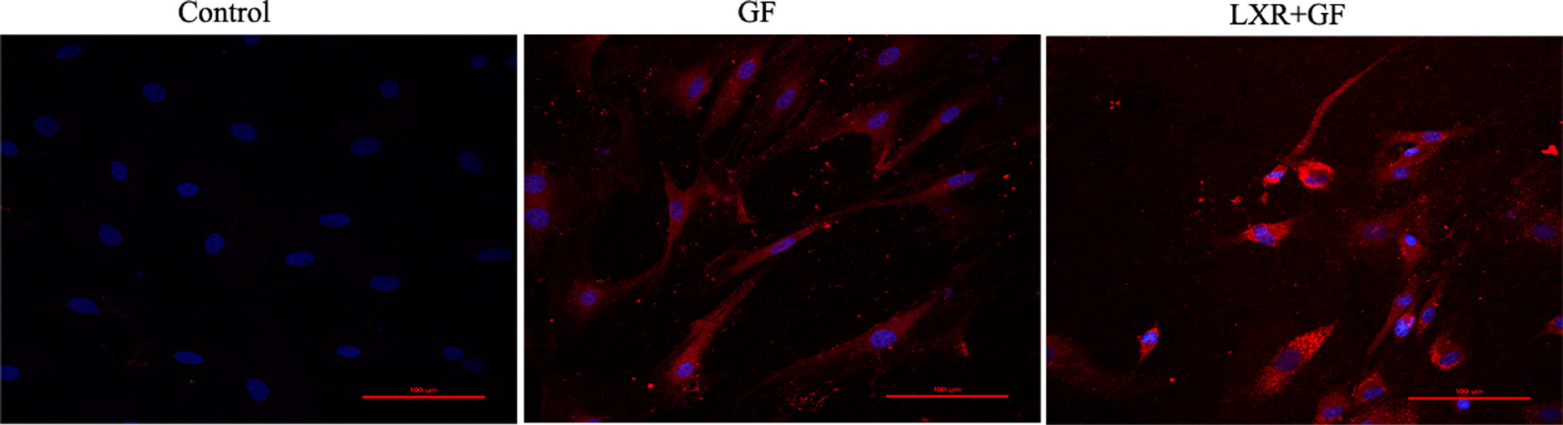
Change in expression of Nestin (×200, Scale bars = 100 μm) No expression of Nestin was detected in control group. Cells in GF group and LXR+GF group displayed single staining with Nestin (dylight 549, red).

Cells in control group probed with Nestin antibody was negative and only show the nuclear stain DAPI. Cells in GF group and LXR+GF group displayed single staining with Nestin (dylight 549, red) (Figure 6).

### Change in the amount of TH positive neurons

**Figure 7:**
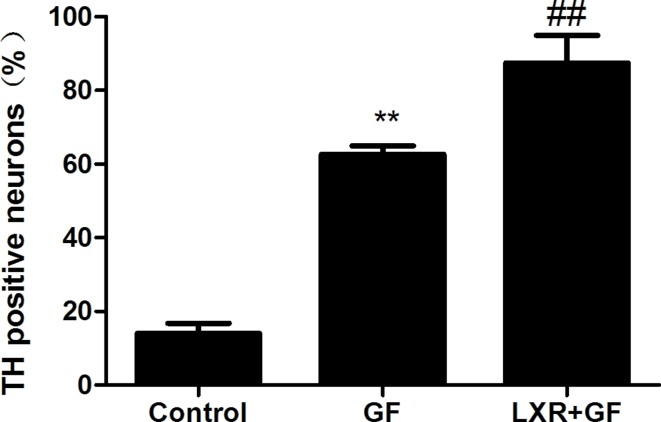
Change in the amount of TH positive neurons Data are expressed as mean ± SD (*n =* 3). ^**^P < 0.01, compared with control group. ^##^*P* < 0.01, compared with GF group.

Quantitative cell count analyses of immunocytochemistry experiments were performed to evaluate the amount of TH positive neurons. Almost cells in control group probed with TH and Tuj1 antibodies were negative and only show the nuclear stain DAPI (according to Figure 3C). Compared with control group, of a total of Tuj1 positive cells counted, 62.61 ± 2.334% of these cells were positive for TH (according to Figure 3C) and the amount of TH positive neurons was significantly increased in GF group (^**^P < 0.01). Compared with GF group, of a total of Tuj1 positive cells counted, 87.42 ± 7.573% of these cells were positive for TH (according to Figure 3C) and the amount of TH positive neurons was significantly increased in LXR+GF group (^##^*P* < 0.01) (Figure 7).

### Effects of alone use of LXR agonist on differentiation of rat BMSCs into DA neurons

**Figure 8:**
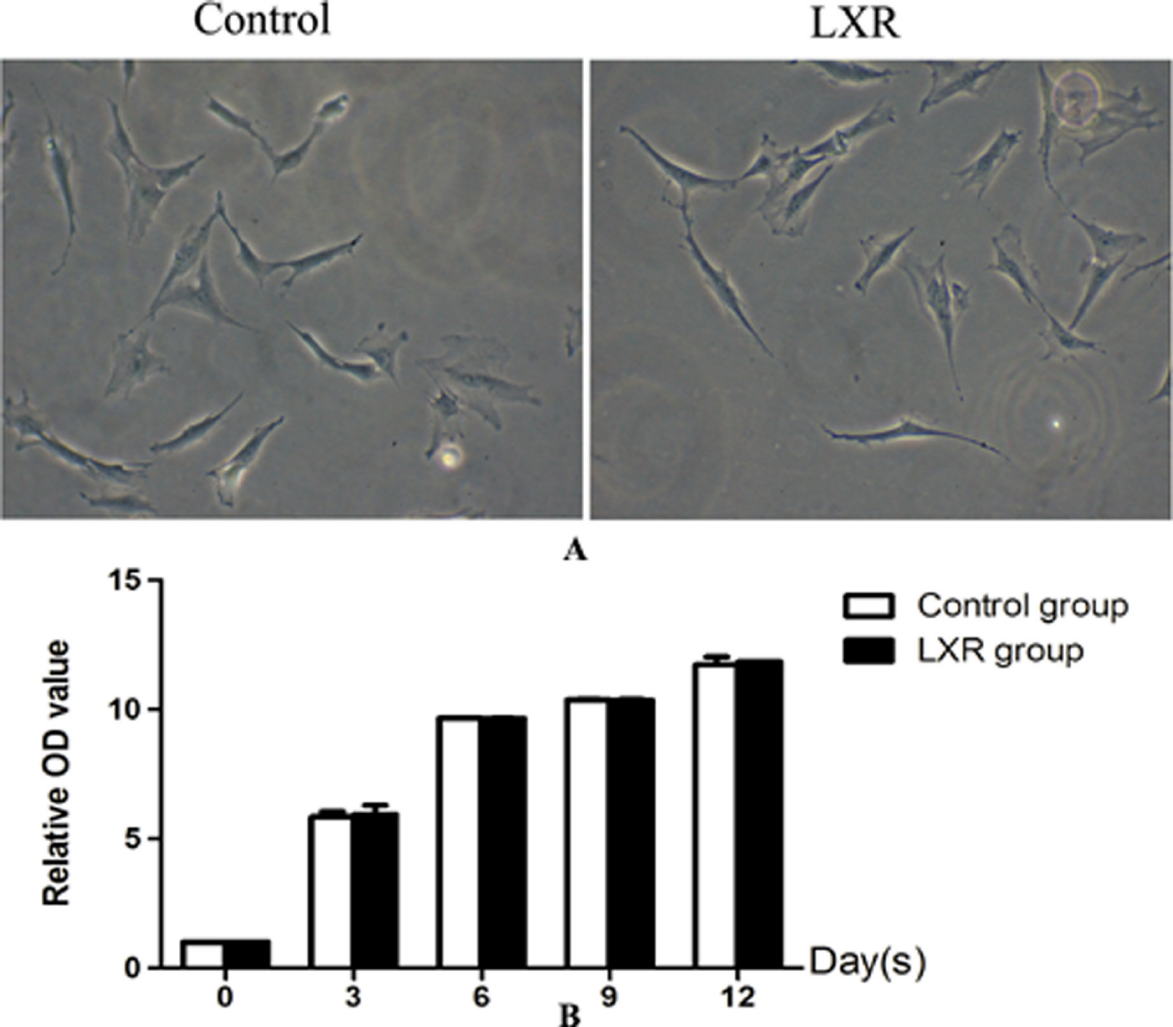
Effects of alone use of LXR agonist on differentiation of rat BMSCs into DA neurons (**A**) Cells in control group and LXR group were typical BMSCs morphology i.e. very flat, symmetrical, and spindle-shaped. (**B**) The growth rate in both control group and LXR group was increased gradually and without significant difference between the two groups.

Cells in control group and LXR group were typical BMSCs morphology i.e. very flat, symmetrical, and spindle-shaped. The growth rate in both control group and LXR group was increased gradually and without significant difference between the two groups. The results indicated that cells treated with LXR agonist alone had no effect on cell proliferation and differentiation and thus LXR group was discussed no more in following research (Figure 8).

### Changes in expressions of LXR

**Figure 9:**
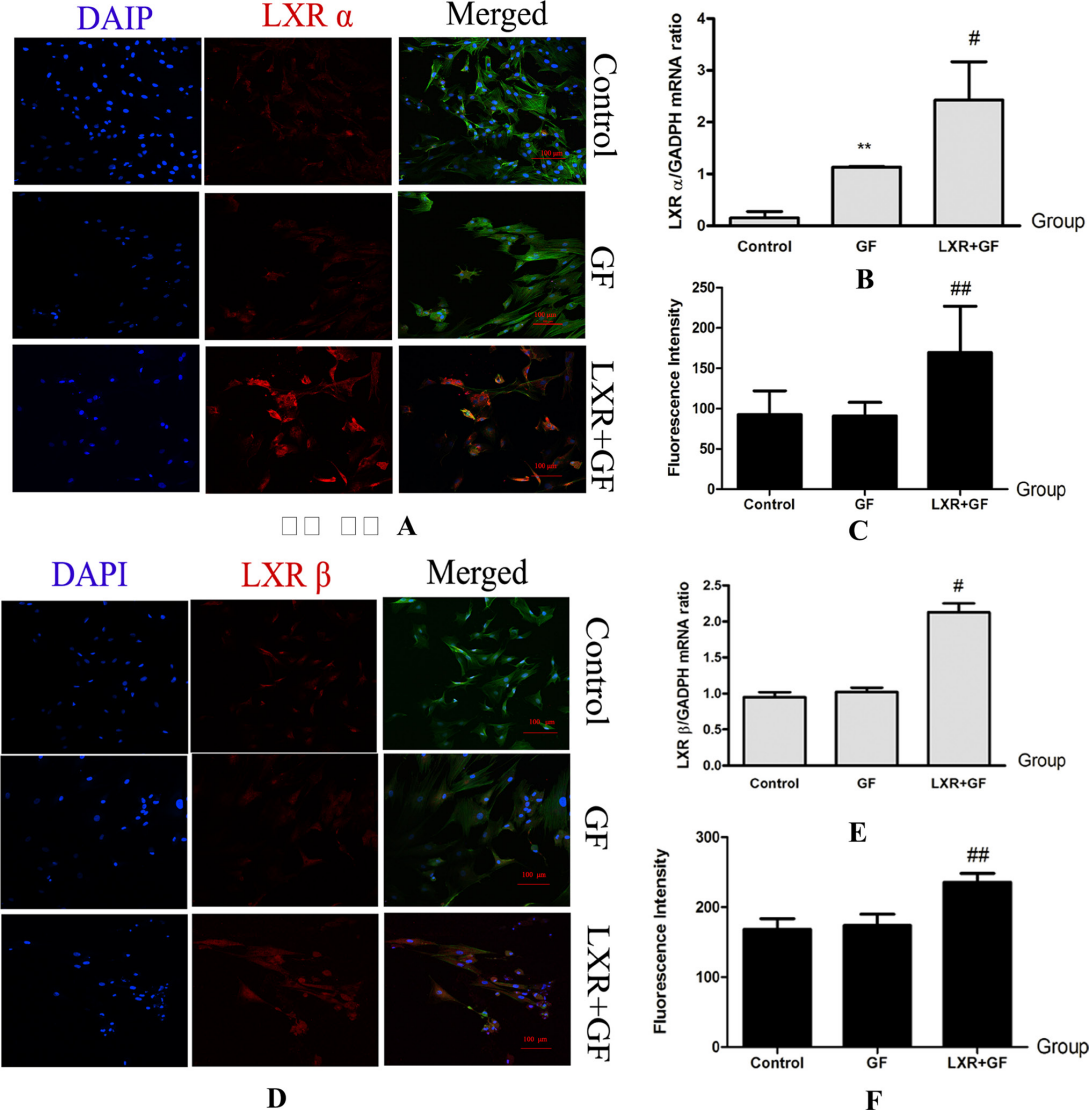
Changes in expressions of LXR (× 200, Scale bars = 100 μm) (**A**–**C**) Changes in expressions of LXR α protein and mRNA. Cells in control group, GF group and LXR+GF group displays merged images of double staining with F-actin (FITC, green) and LXR α (dylight 549, red). Colocalization was identified by the yellow fluorescence in the merged images. Group data showing changes in expressions of LXR α protein and mRNA. Data are expressed as mean ± SD (*n =* 3–5). ^**^P < 0.01, compared with control group; ^#^*P* < 0.05 and ^##^*P* < 0.01, compared with GF group, respectively. (**D**–**F**) Changes in expressions of LXR β protein and mRNA. Cells in control group, GF group and LXR+GF group displays merged images of double staining with F-actin (FITC, green) and LXR β (dylight 549, red). Colocalization was identified by the yellow fluorescence in the merged images. Group data showing change in expressions of LXR β protein and mRNA. Data are expressed as mean ± SD (*n =* 3–5). ^#^*P* < 0.05 and ^##^*P* < 0.01, compared with GF group, respectively.

Compared with control group, expression of LXR α mRNA was significantly increased in GF group (^**^P < 0.01). Compared with GF group, mRNA and protein expressions of LXR α and LXR β were significantly increased in LXR+GF group (^#^*P* < 0.05 and ^##^*P* < 0.01, respectively) (Figure 9).

### Changes in expressions of DA development-related genes

**Figure 10:**
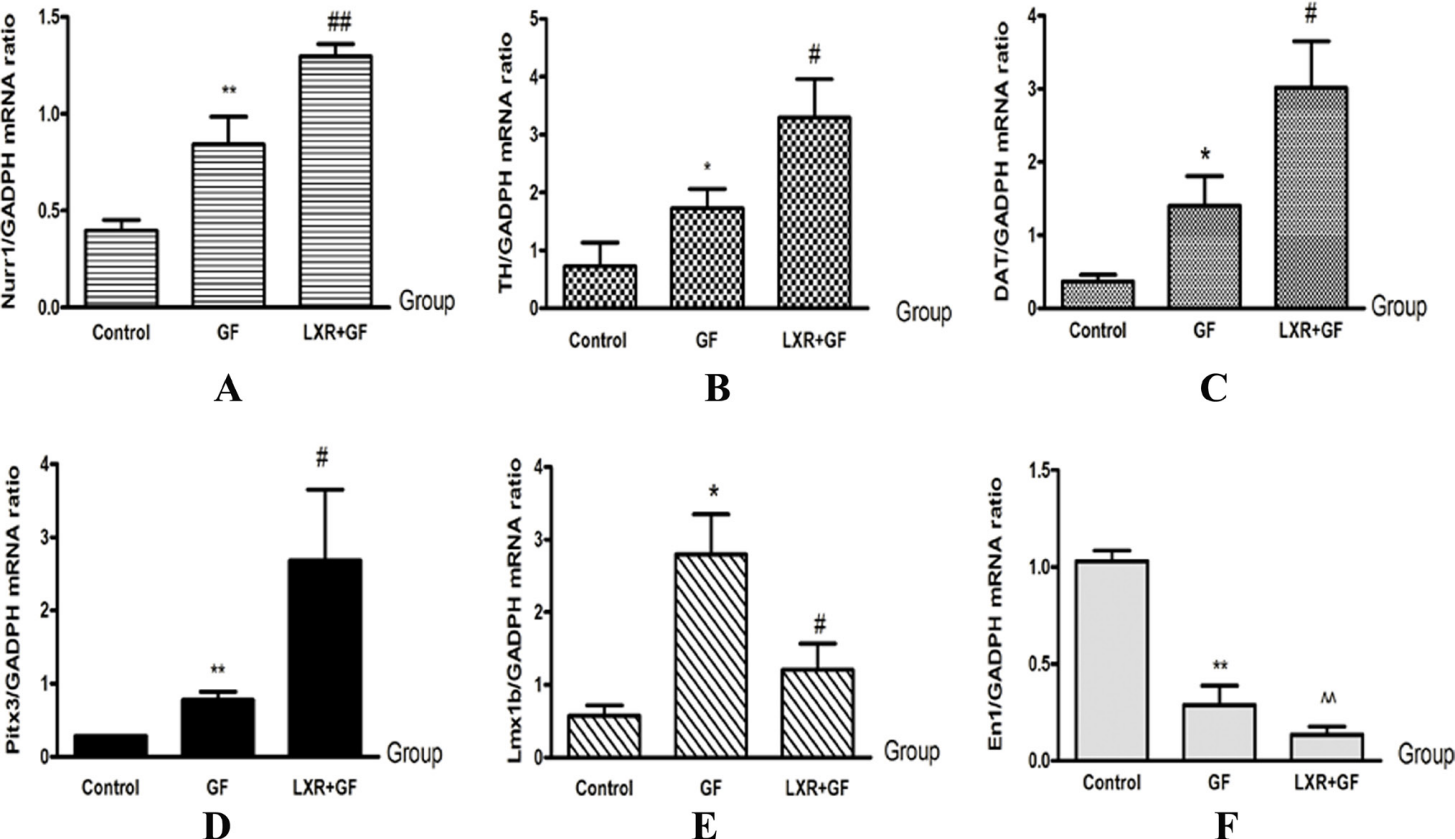
Change in expressions of DA development-related genes (**A**–**F**). Group data showing change in expressions of DA development-related genes. Data are expressed as mean ± SD (*n =* 3). ^*^P < 0.05 and ^**^P < 0.01, compared with control group, respectively. ^#^*P* < 0.05 and ^##^*P* < 0.01, compared with GF group, respectively. ^^^^*P* < 0.01, compared with control group.

GF group showed significantly higher levels of TH, DAT, Nurr1 and Pitx3 mRNA compared with control group (^*^P < 0.05 and ^**^P < 0.01, repectively). LXR+GF group express significantly higher levels of TH, DAT, Nurr1 and Pitx3 mRNA compared with GF group (^#^*P* < 0.05 and ^##^*P* < 0.01, repectively). Compared with control group, expression of En1 was decreased significantly in GF group (^**^P < 0.01). Compared with control group, expression of En1 was decreased significantly in LXR+GF group (^^^^*P* < 0.01). Compared with control group, expression of Lmx1b was increased significantly in GF group (^*^P < 0.05). Compared with GF group, expression of Lmx1b was decreased significantly in LXR+GF group (^#^*P* < 0.05) (Figure 10).

## DISCUSSION

In the present study, we found several important findings. First, on the basis induction of GF, LXR activation leads to improve induction efficiency and shorten induction period of rat BMSCs into DA neuron-like cells. Second, the mechanism of LXR agonist promoting differentiation of rat BMSCs into DA neuron-like cells may be involved in regulating DA development-related genes expressions by LXR activation. Third, LXR agonist alone did not induce the differentiation of rat BMSCs into DA neurons.

DA neurons derived from stem cells are a valuable source for CRT in PD, to study the molecular mechanisms of DA neuron development and to screen pharmaceutical compounds that target DA disorders [23]. Compared with other stem cells, BMSCs have significant advantages and greater potential for immediate clinical application. Much effort has been laid to investigate the appropriate *in vitro* cues which drive BMSCs to differentiate into DA neurons. In recent years, the efficiency of DA neuron generation from BMSCs has been reported variation ranging from 12.7% to 67% and the time for period of induction has shown variation ranging from 12 to 21 days [7, 24–29].

Use of GF as inducers of differentiation has led to the generation of better results in terms of efficiency, cytotoxicity, and long term maintenance of these cells in culture, instead of chemicals [30]. The development of DA neurons in the embryo is dependent on the interaction of two GF-SHH and FGF8 [31]. Additionally, bFGF promotes neurogenesis and enhances differentiation and survival of DA neurons [32]. Thus, BMSCs treated with GF is reliable method [7].

In the present study, the effects of LXR agonist on DA neurons’ differentiation were investigated. We found that the growth rate of cells in GF group and LXR+GF group was increased and later decreased. Consistent with the results of Trzaska KA *et al*, both cells in GF group and LXR+GF group revealed the appearance of extended long cellular processes and retracted cell bodies, which were the typical neuronal morphology. The results indicated that rat BMSCs treated with GF alone or LXR agonist plus GF leads to directed neuronal differentiation and decreased cell proliferation. Moreover, cells in GF group were induced to TH positive neurons with 62.61% efficiency in 12 days of period of induction, which is similar to the results of Trzaska KA *et al.* [7]. In comparison with GF group, our experimental results showed that cells in LXR+GF group were induced to TH positive neurons with 87.42% of efficiency in 6 days of period of induction. The optimum protocol is that simultaneously combined utilization of LXR (0.5 μM) agonist with GF with a period of induction in 6 days. These results together indicated that simultaneous addition of LXR agonist and GF could significantly improve induction efficiency and shorten induction period of rat BMSCs differentiation into DA neuron-like cells. These results also suggest that LXR may be involved in the mechanisms of rat BMSCs differentiation into DA neuron-like cells. Similarly, oxycholesterol (brain endogenous LXR ligands) treatment of human ESCs during DA differentiation could increase neurogenesis and the number of mature DA neurons [20]. The maximal neurogenic effects of 22-hydroxycholesterol were reached at concentration of 0.1–0.5 μM, while the toxicity dosage of 22-hydroxycholesterol beyonds 5 μM [20]. Maximal expressions were obtained at simultaneous addition of LXR agonist and GF (Day0). It indicated that early activation of LXR contributes to DA neurons’ differentiation.

The *in vivo* ontogeny of DA neurons has been extensively studied, but many of the mechanisms and factors involved remains unclear. LXR are critical for VM neurogenesis *in vivo* and developmental midbrain neurogenesis is decreased in LXR double knockouts [20, 33]. Moreover, activation of LXR is important for *in vivo* development as well as sufficient for inducing ESCs to differentiate into DA neurons [20, 21]. The present study showed that, compared to control group, GF increased the expression of LXR α mRNA and the efficiency of induction, interestingly, and the co-administration of LXR agonist and GF further increased LXR α mRNA and protein expressions and accompanied higher efficiency. In the same time, expressions of LXR β mRNA and protein were significantly also increased. These results indicated that LXR activation upregulated expression of LXR. Furthermore, these results also indicated that not only LXR β but also LXR α activation contributes to the differentiation of rat BMSCs into DA neuron-like cells and co-expression of LXR α and LXR β may lead to improved efficiency and shorten induction period of rat BMSCs into DA neuron-like cells. Previously, under the action of GF, ESCs can differentiate into neuronal progenitors which can further differentiate into DA neurons under action of the LXR agonist and other factors [34]. However, further studies are needed to explore the relationship between LXR activation/overexpress and DA neurons differentiation.

Previous studies showed that many transcription factors, including TH, DAT, En1, Lmx1b, Nurr1, and Pitx3, have provided important results on the mechanism of controlling identity, growth and survival of neuronal precursors [13, 35, 36]. Our experimental results also showed that the expression of these DA development-related genes including TH, DAT, Nurr1 and Pitx3 were increased in both GF group and LXR+GF group, especially in LXR+GF group, which is similar to the results of Trzaska KA *et al.* [7]. Salemeh *et al.* found that overexpression of Nurr1 and Pitx3 in induced Pluripotent Stem Cells (iPSCs) could efficiently program iPSCs into functional DA neuron-like cells [37]. The expressions of En1 and Lmx1b were significantly decreased in GF group and LXR+GF group, especially in LXR+GF group. Fan LX *et al.* also reported that the expression of En1 was decreased with during-dependently differentiation of human bone marrow multipotent stem cells into DA neurons [24]. These results together indicated that changes in expressions of DA development-related genes, including TH, DAT, En1, Lmx1b, Nurr1, and Pitx3, which may contribute to differentiation of rat BMSCs into DA neuron-like cells. Moreover, as shown in the ‘Results’, induction period of LXR agonist had obviously effects on expressions of Tuj1, TH and DA development-related genes and the effects remains unclear. Thus, we will verify the important micro-environmental influences in DA neuron development and survival in future studies.

In the present study, cells in control group and LXR group were typical rat BMSCs morphology and cells in control group expressed neither early neuronal marker nestin nor mature neuronal marker Tuj1. The growth rate in both control group and LXR group was increased gradually and without difference between the two groups. These results together suggested that single use of LXR agonist had no effect on cell proliferation and differentiation. It indicated that LXR agonist alone cannot directly induce BMSCs differentiation into the DA neurons.

In conclusion, our experimental results indicated that, on the basis induction of GF, LXR agonist promotes differentiation of rat BMSCs into DA neuron-like cells and the mechanism may be involved in regulating DA development-related genes expressions by LXR activation. Our experimental results suggest that LXR can be considered as a candidate target for drug development to improve the differentiation of rat BMSCs into DA neurons. So, the survival of BMSC-derived DA neuron-like cells and dopamine release after transplantation is worthy of further study.

## MATERIALS AND METHODS

Ethics statement: All the experimental procedures were approved by the Animal Laboratory Administrative Center and the Institutional Ethics Committee at Chongqing Medical University and also in accordance with the National Institutes of Health guidelines.

### Isolation and culture of rat BMSCs

Rat BMSCs were isolated and harvested from bone marrow of the tibias and femur of 4-weekth-old male Sprague–Dawley rats by inserting a 21-ga needle into the shaft of the bone and flushing it with 30 ml Dulbecco’s modified Eagle/F12 medium (DMEM/F-12; Hyclone, USA) supplemented with 20% fetal bovine serum (FBS; Gibco, Grand Island, NY, USA). Cells were grown in a culture flask and cultured under a routine condition (37°C, air plus 5% CO_2_) for 48 h. Non-adherent cells were removed by changing the medium and the resulting monolayer of cells was trypsinised. Since 1st passage, cells were cultured in DMEM/F-12 supplemented with 10% FBS. Aliquots were cultured further, or frozen and stored. For all the subsequent experiments, cells from 3rd passage were used [22].

### Identification of BMSCs by the method of flow cytometry

According to the manufacturer’s instructions, CD44-PE, CD29-FITC, CD90-PE, CD11b-PE or CD45-FITC rat-specific antibodies (eBioscience, USA) was added to the bottom of tubes, after which 100 μl (6×10^6^ cells/μl) of a single-cell suspension of cultured rat BMSCs was added. The mixture was incubated for 30 min at room temperature in the dark and washed [22]. Positive cells were detected by flow cytometry (Becton-Dickinson, USA). Rat IgG1-FITC and IgG1-PE (eBioscience, USA) were used as isotype controls.

### Induction differentiation of rat BMSCs

Rat BMSCs were divided into 4 groups and plated onto Costar 24 well plates where each plate contained 2.0×10^5^ cells and cells were cultured at 37°C with 5% CO2. ①Control group: Cells were cultured in DMEM/F-12 supplemented with 10% FBS (Figure 11①).

**Figure 11:**
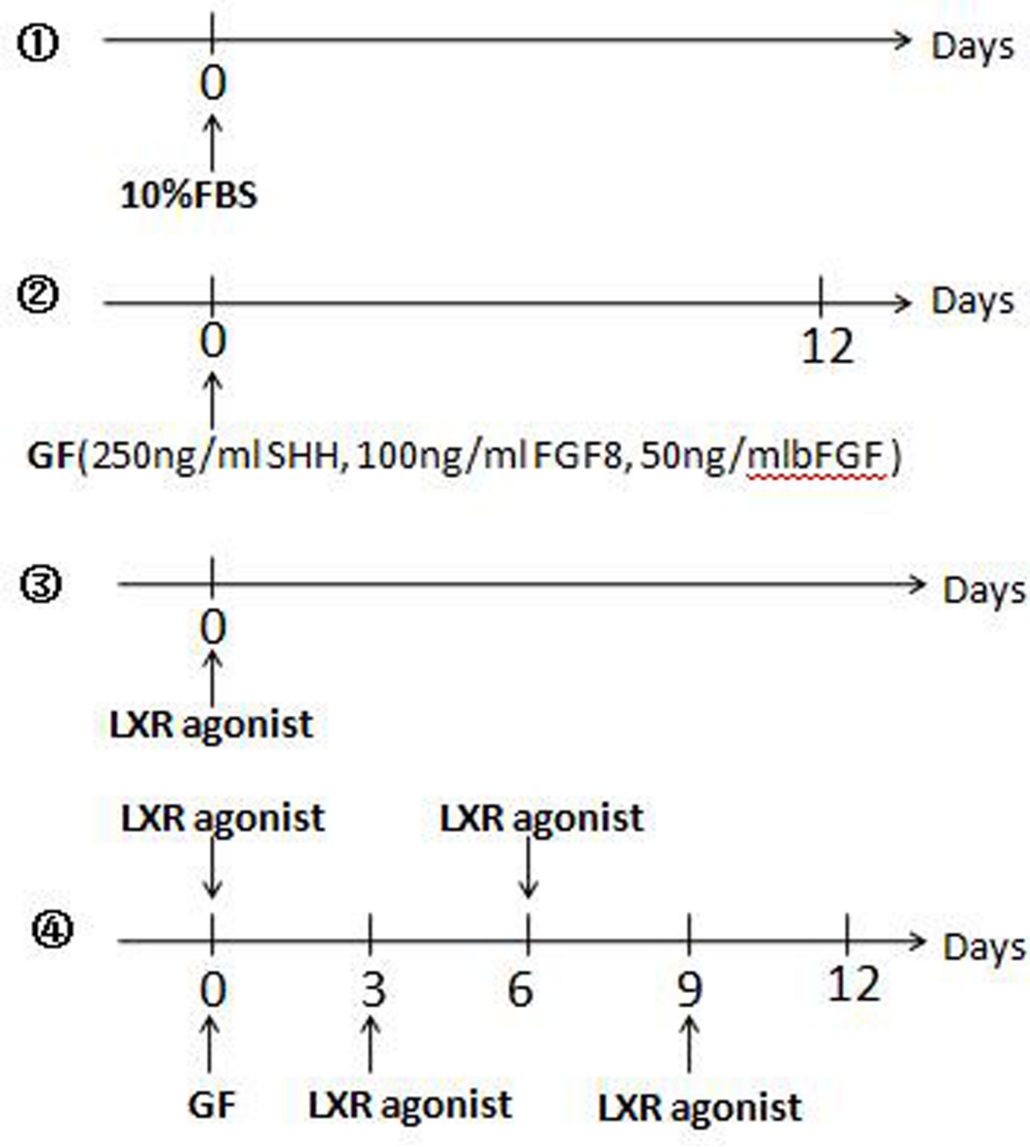
Induction differentiation of rat BMSCs ①Control group: Cells were cultured in DMEM/F-12 supplemented with 10% FBS. ②GF group: The cells were induced only once with a cocktail of 250 ng/ml Recombinant Murine SHH, 100 ng/ml Recombinant Human FGF8, and 50 ng/ml Recombinant Rat basic-FGF. The medium was not replaced in 12 days. ③LXR group: Cells were cultured in DMEM/F-12 supplemented with 10% FBS and 0.5 μM LXR agonist was added into the medium. ④Determine the adding time of LXR agonist in LXR+GF group: Based on the method of GF group, 0.5 μM LXR agonist was added into the medium at 3-day intervals respectively.

②Growth factors treated group (GF): DMEM/F-12 containing 10% FBS was replaced with Neurobasal medium (Invitrogen/Gibco, USA) and B27 supplement (Invitrogen/Gibco, USA) in the next day. The 50× (100%) B27 supplement was diluted to a final concentration of 0.25× (0.5%). The cells were induced only once with a cocktail of 250 ng/ml Recombinant Murine SHH (PeproTech, Rocky Hill, NJ, USA), 100 ng/ml Recombinant Human FGF8 (PeproTech, Rocky Hill, NJ, USA), and 50 ng/ml Recombinant Rat basic-FGF (bFGF; PeproTech, Rocky Hill, NJ, USA). The medium was not replaced in 12 days [7] (Figure 11②).

③LXR agonist group (LXR): Cells were cultured in DMEM/F-12 supplemented with 10% FBS and 0.5 μM LXR agonist was added into the medium (Figure 11③).

④LXR agonist (TO901317) and growth factors treated group (LXR+GF): On the basis induction of GF, effects of LXR agonist on differentiation of rat BMSCs into DA neurons in the time-dependent and concentration-dependent manner were investigated. According to the results of (a) (b) and (c), the cells were induced only once with the cocktail and the medium was not replaced during the induction period.

Determine the adding time of LXR agonist: Based on the method of GF group, 0.5 μM LXR agonist (Sigma, St. Louis, MO, USA) was added into the medium at 3-day intervals respectively (Figure 11④).

Determine the time for induction period of LXR agonist: 0.5 μM LXR agonist was added into the medium according to the result of (a) and cells’ status were observed at 3-day intervals.

Determine the concentration of LXR agonist: Different concentrations of LXR agonist (0.125, 0.25, 0.5, 1 and 2 μM) were added into the medium according to the results of (a) and (b).

### Cell counting kit-8

A Cell Counting Kit-8 (CCK-8; Dojindo, Tokyo, Japan) assay was used to test the growth rate following the manufacturer’s instructions. 3000 cells/well of rat BMSCs were seeded into a 96-well plate and allowed to adhere overnight [38]. At 3-day intervals, the growth rate for each group was measured by application of CCK-8 kit and optical density (OD) was determined at 450 nm using a microplate reader (Thermo, Finland).

### Immunocytochemical assay

Cells were fixed with 4% paraformaldehyde and blocked with 5% bovine serum albumin in PBS. Cells were stained with primary antibodies overnight at 4°C. Dilutions were as follows: Nestin, 1:100 (Abcam, Cambridge, UK); TH, 1:200 (Millipore, CA, USA); β III tubulin (Tuj1; Millipore, CA, USA), 1:200 (Millipore, CA, USA); LXR α receptor, 1:200 (Abcam, Cambridge, UK); LXR β receptor, 1:200 (Abcam, Cambridge, UK). Appropriate secondary antibodies anti-rabbit IgG-dylight 549(Abbkine, CA, USA) and anti-mouse IgG-dylight 488(Abbkine, CA, USA); nuclear stain 4, 6-diamidino-2-phenylindole (DAPI; Beyotime Biotechnology, Shanghai, China); and cytoskeletal stain Texas Red phalloidin (F-actin; Molecular Probes, Eugene, OR, http://probes.invitrogen.com) were used for detection and visualization [7]. Finally, the numbers of total cells and TH/Tuj1 positive cells were determined using a laser scanning confocal microscope (Nikon A1*R, Japan) and fluorescence intensity was analysis by NIS-Elements AR 4.2 (Nikon, Japan).

### Quantitative real-time PCR (qPCR)

Total RNA was extracted from the proximal femur using RNAiso Plus (Total RNA Extraction Reagent, TaKaRa Bio Inc, Tokyo, Japan) in accordance with the manufacturer’s protocol. Total RNA of each group was extracted at optimal effect of induction. Approximately 1000ng of total RNA was reverse transcribed using the Prime Script1TM RT Reagent Kit with gDNA Erase (TaKaRa Bio Inc, Tokyo, Japan). qPCR was performed in triplicate in a 20μl volume, using SYBR1Premix Ex TaqTM II (TaKaRa Bio Inc, Tokyo, Japan) and the CFX96 Touch TM Real-Time PCR Detection System (Bio-Rad, CA, USA) according to the manufacturers’ instructions. Gene expression was determined relative to the housekeeping gene GAPDH using the 2^–ΔΔCt^ method [39]. Specific primers were list as follows within the Sangon Biotech (Shanghai, China) designed (Table 1).

**Table 1:** Polymerase chain reaction primer pairs

Gene	GenBank accession number	Primer sequence	Anneal (°C)
*TH*	NM_012740.3	5′ TTCTGGAACGGTACTGTGG 3′ 5′ AATCACGGGCGGACAGTA 3′	60
*Nurr1*	NM_019328.3	5′ AGAGGGTGGGCAGAGAAGAT 3′ 5′ CTGGGTTGGACCTGTATGCT 3′	60
*DAT*	NM_012694.2	5′TCCTGAAAGGTGTGGGCT3′ 5′GAGCAGTTGGGGCTATTC3′	60
*En1*	XM_001056699.6	5′ GGAGAAAGACTCGGACAGGT3′ 5′ GGTCGTAAGCAGTTTGGCTA 3′	60
*Pitx3*	NM_019247.1	5′ GAATCGCTACCCCGACATGA 3′ 5′ TACACCTCCTCGTAGGGTGG 3′	60
*Lmx1b*	NM_053709.1	5′ TGCGGAAGGGTGATGAGTTC 3′ 5′ CCACTGCCCTTATTCTGGCT 3′	60
*Nr1h3*	NM_031627.2	5′ TTCGTCCACAGAAGCGGAAA 3′ 5′ CGTGCTCCCTTGATGACACT 3′	60
*Nr1h2*	NM_031626.1	5′ AGCGATCTTTCTCCGACCAG 3′ 5′ TTGGCGAAGTCCACGATCTC 3′	60
*GAPDH*	NM_017008.4	5′ ACAGCAACAGGGTGGTGGAC3′ 5′ TTTGAGGGTGCAGCGAACTT 3′	60

### Statistical analyses

The results are expressed as the means ± standard deviation (SD) and were analyzed using SPSS 17.0 (SPSS Inc. Chicago, USA). The data’s statistical analyses were performed using a one-way analysis of variance (ANOVA) test. *P*-values < 0.05 were considered significant.
